# Mineral Acquisition from Clay by Budongo Forest Chimpanzees

**DOI:** 10.1371/journal.pone.0134075

**Published:** 2015-07-28

**Authors:** Vernon Reynolds, Andrew W. Lloyd, Christopher J. English, Peter Lyons, Howard Dodd, Catherine Hobaiter, Nicholas Newton-Fisher, Caroline Mullins, Noemie Lamon, Anne Marijke Schel, Brittany Fallon

**Affiliations:** 1 School of Anthropology, Oxford University, Oxford, United Kingdom; 2 School of Environment & Technology, University of Brighton, Brighton, United Kingdom; 3 Budongo Conservation Field Station, Masindi, Uganda; 4 School of Psychology, St Andrews University, St Andrews, Scotland; 5 School of Anthropology and Conservation, University of Kent, Canterbury, United Kingdom; 6 Institute of Biology, University of Neuchâtel, Neuchâtel, Switzerland; 7 Animal Ecology, Utrecht University, Utrecht, The Netherlands; Institut Pluridisciplinaire Hubert Curien, FRANCE

## Abstract

Chimpanzees of the Sonso community, Budongo Forest, Uganda were observed eating clay and drinking clay-water from waterholes. We show that clay, clay-rich water, and clay obtained with leaf sponges, provide a range of minerals in different concentrations. The presence of aluminium in the clay consumed indicates that it takes the form of kaolinite. We discuss the contribution of clay geophagy to the mineral intake of the Sonso chimpanzees and show that clay eaten using leaf sponges is particularly rich in minerals. We show that termite mound soil, also regularly consumed, is rich in minerals. We discuss the frequency of clay and termite soil geophagy in the context of the disappearance from Budongo Forest of a formerly rich source of minerals, the decaying pith of *Raphia farinifera* palms.

## Introduction

The chimpanzees (*Pan troglodytes schweinfurthii*) of the Sonso community in the Budongo Forest Reserve, Western Uganda, have been studied continuously since 1990. Their diet consists in the main (80% or more) of fruits and leaves, supplemented by flowers, bark, insects, and meat [1]. In recent years they have increasingly been seen eating clay from clay pits, and drinking clay in suspension in water from holes under trees. The clay soil at these locations has a lighter, greyer colour than the red-brown soil of the forest generally. The clay suspended in water (‘clay-water’) can be drunk directly from the source, but is mainly obtained by the use of clay “sponges”, leaves of *Acalypha* and other tree species which are chewed in the mouth, removed by hand, dipped in the clay-water, and then returned to the mouth where the liquid contents are squeezed out with the tongue, after which the same sponge may be used again or replaced with a new one.

In this paper we analyse the mineral contents of samples of clay, clay-rich water, and clay obtained with the use of leaf sponges. The analysis shows that clay consumption provides the Sonso chimpanzees with a number of essential minerals. We also compare the mineral content of clay with that of termite mound soil, which is also eaten by the chimpanzees, and forest soil that is not eaten by Budongo chimpanzees. Finally we compare the mineral content of clay with that of the decaying pith of a forest palm tree: *Raphia farinifera* (RF). Decaying RF pith formerly provided the Sonso chimpanzees with minerals, in particular sodium [2] but owing to anthropogenic destruction has now all but disappeared from the forest. Here we investigate whether the increased frequency of observations of clay feeding in recent years can be linked with the decline of RF palms.

## Materials and Methods

### Field collection of samples

All samples were collected by members of the Budongo Conservation Field Station (BCFS) from within the Budongo Forest Reserve. Clay samples were collected using sterile gloves and a clean knife from inside clay holes, in some cases under water, and placed in new plastic or glass collection tubes over silica beads. Samples of clay leaf sponges discarded by chimpanzees were picked up from the ground using sterile gloves while still wet and stored over silica beads in glass tubes. Ground soil was cut with a clean knife from 10cm below the surface of leaf litter. Pieces of termite mound soil, where chimpanzees had been seen feeding, were cut from 2 *Macrotermes sp*. mounds and one *Pseudacanthotermes sp*. mound with a clean knife. Soil samples were placed in glass tubes and in the field station the screwcap of the tubes was removed and the samples air-dried at room temperature. Samples of clay-water, water that occurs in puddles on the ground (ground water) and river water were collected by dipping clean glass or plastic collection tubes into water. All samples were transported to BCFS in clean plastic bottles, with care taken to avoid all skin contact. Sample bottles were shaken to ensure even distribution of any suspension and 10ml samples were transferred to unused glass or plastic tubes whilst wearing gloves. All samples were labelled with date and location of collection site (100x100m BCFS grid reference) and sent to the UK for laboratory analysis.

### Laboratory analysis of samples

The samples of clay, leaf sponges, termite mound soil and ground soil were dried to constant weight in an oven at 105°C and then ashed at 450°C for 6 hours. The total mass of the dried material was determined. 0.1g of the material was weighed into a 10ml centrifuge tube. 3ml of Aqua Regia was added before digesting all samples in a water bath at 85°C for 3 hours. 7ml of ultrapure Type 1 water was added and all samples were mixed using a vortex mixer. 1ml was decanted from each sample and diluted 1:10 with Type 1 water for analysis. In the case of water samples, sample tubes were homogenised with a vortex mixer, and 1ml was decanted and made up to 10ml with 3% HNO_3_ and left for 3 hours. The elemental content of each sample was then determined using a Perkin Elmer Optima 2100 DV Inductively Coupled Plasma Optical Emission Spectrometer (ICP-OES). Standards and a blank were made up at 2, 4, 6, 8 and 10 ppm concentrations with 3% HNO_3_ and three replicates of each element were measured. The elemental content per kg of dried material was calculated from the raw data. Chloride (*Cl*) measurements were made using a chloride ion selective electrode (solid AgCl/Ag_2_S matrix) using a calomel reference electrode with saturated KNO_3_ bridge. Calibration standards were made up from 1000ppm Cl-stock (1.6462 g/l). The small volumes of solutions of the samples were put into a watch glass to facilitate the insertion of the electrodes.

### Other data sources

Besides field and laboratory analysis of water, clay-water, clay and termite mound samples, data from previous studies were incorporated into the analysis in order to compare recent and past feeding on clay, feeding on the decaying pith of *Raphia farinifera*, a forest palm tree (RF), feeding on the decaying wood from a forest tree *Cleistopholis patens* (CP), and other species that provide food for chimpanzees. These data were obtained from the BCFS database, records supplied by present and past researchers, and BCFS field assistants.

### Non-quantitative measures of behaviour frequency

In order to compare the frequency of behaviours in a very different context, that of cultural variation between chimpanzee communities, Whiten et al [3,4] used a non-quantitative set of terms to describe frequency of behaviours. These terms were “customary” for commonly observed behaviours, “habitual” for behaviours observed repeatedly in several individuals, “present” for behaviours clearly identified but neither customary nor habitual, and “absent” for behaviours not recorded [4]. We employ these same terms here in the context of measuring the relative frequency of consumption of potential mineral resources.

### Statistical analysis

All statistical tests were done with SPSS Statistics 22. In order to compare the mineral content of samples, they were divided into 7 groups: clay, clay-water, leaf sponges, control soil, control ground water, control river water, and termite soil. Tests for normality were applied. Data were not normally distributed in any of the groups and therefore non-parametric tests were used to establish whether concentrations of minerals differed between the groups. In addition, statistical comparisons were made with known mineral-rich vegetative samples (RF and CP) described in earlier studies [2,5]. Results of tests were considered statistically significant if they fell below p = 0.05.

## Results

**Fig 1:**
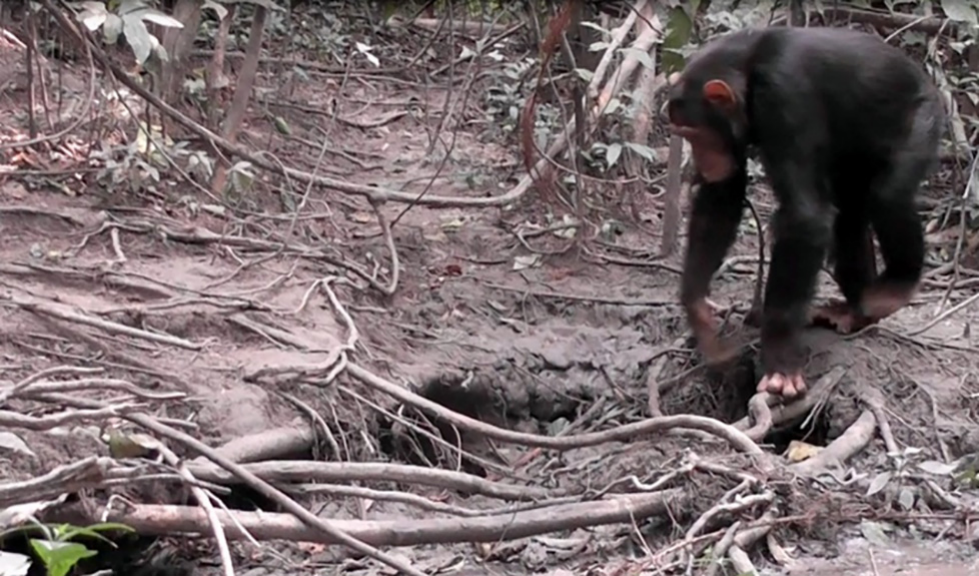
Subadult male chimpanzee Frank approaching clay hole (Photo: A. Schel).

Chimpanzees were observed eating clay directly, drinking clay-water, and obtaining clay-water by use of leaf sponges that were eventually dropped on the ground. The chimpanzees used their fingers to extract the clay directly from the ground, which was then eaten and not spat out. Fig 1 shows a clay hole, approached by a subadult male chimpanzee (FK).

**Fig 2:**
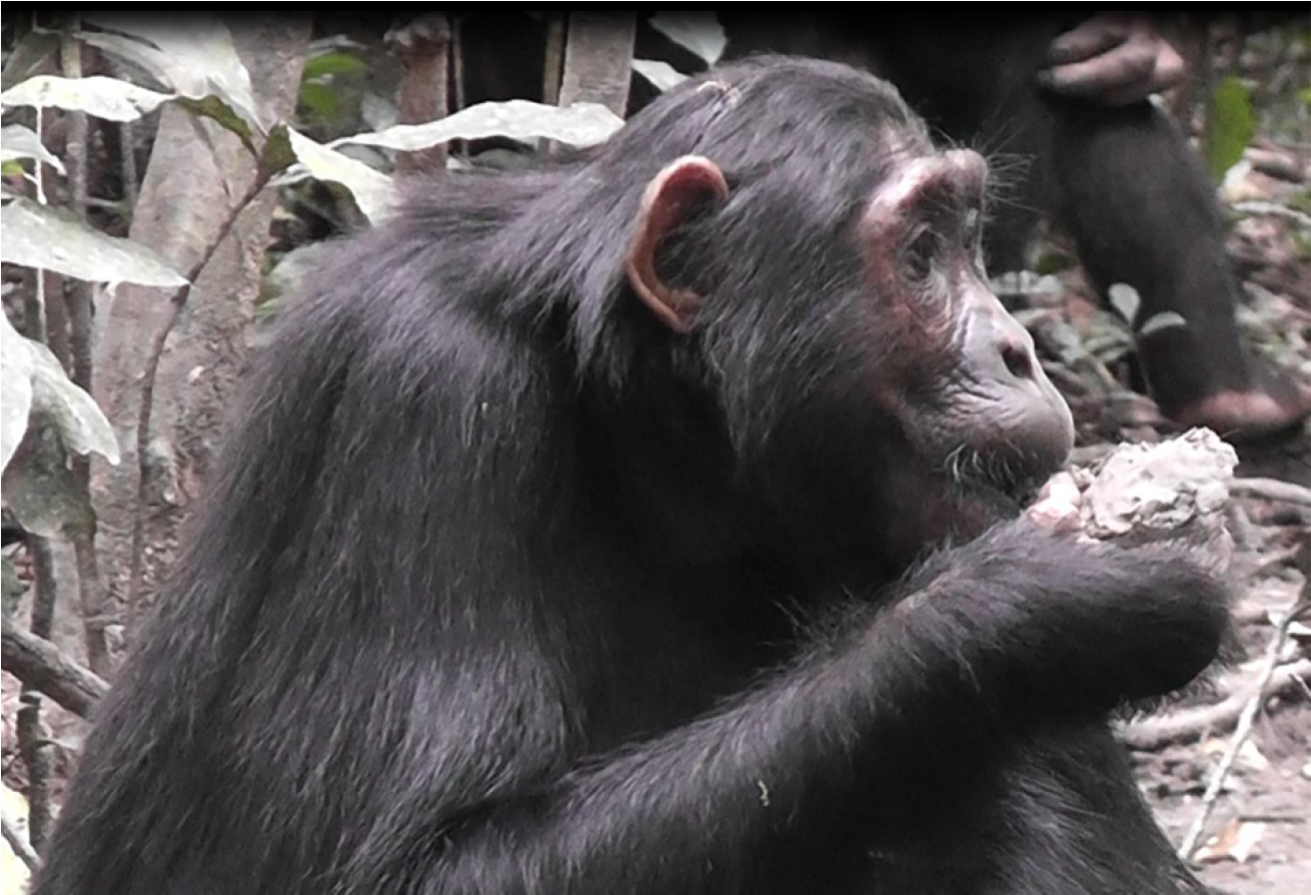
Subadult female Kana eating clay (Photo: A. Schel).

Fig 2 shows a subadult female chimpanzee (KN) eating a lump of clay. Occasionally, leaves were eaten with the clay. Footage of clay eating by a juvenile male (KS) is shown in S1 Video.

**Fig 3:**
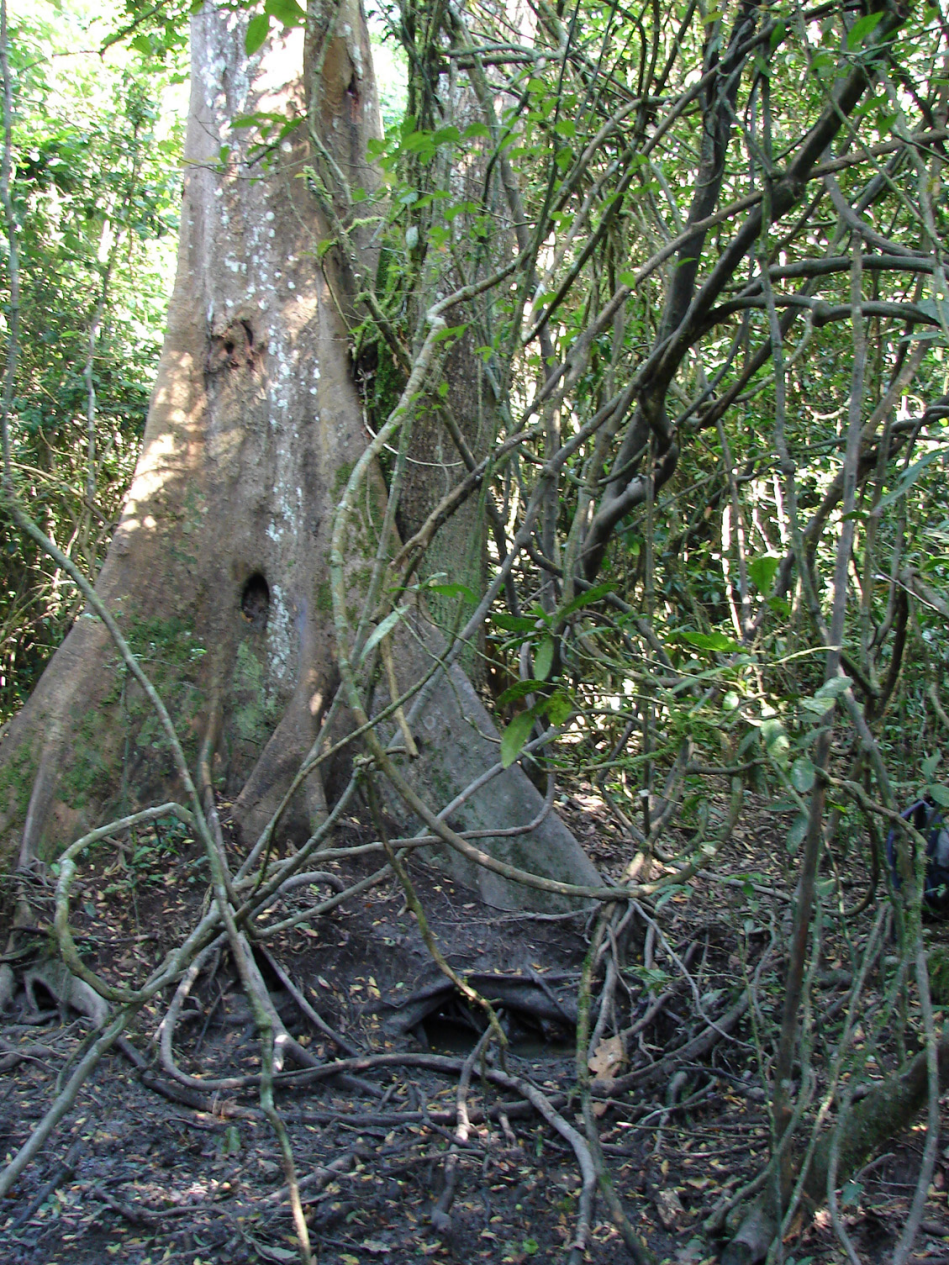
Clay waterhole at base of tree (Photo: V. Reynolds).

**Fig 4:**
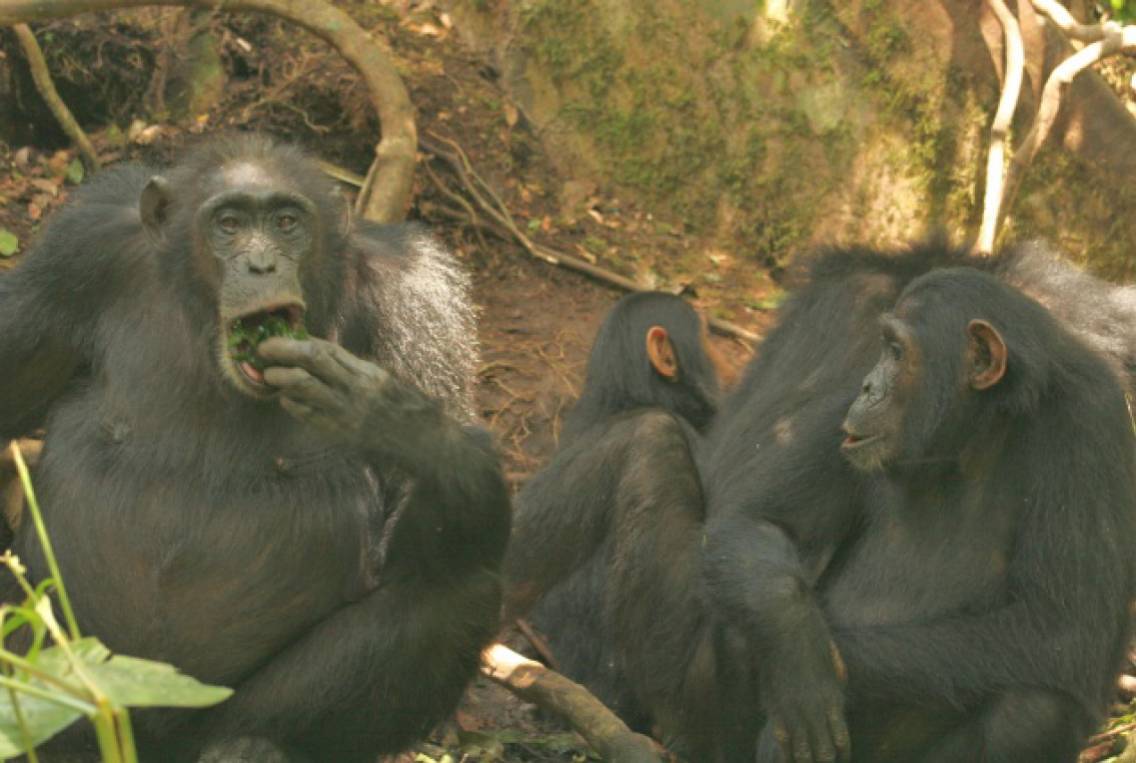
Adult female Nambi (L) using a leaf-sponge at waterhole, watched by 10-yr old juvenile Karo (R) (Photo: C. Hobaiter).

In addition to obtaining clay directly from the clay holes with their fingers, chimpanzees were observed obtaining clay-rich water using leaf sponges and by drinking directly with the mouth (Figs 3 and 4 and S2 Video). This waterhole was located in clay soil below the roots of two adjoining trees of different species, *Cynometra alexandri* and *Mimusops bagshawei*. The leaf sponges were dipped into the water, placed in the mouth, and sucked or chewed before being discarded.

The results of laboratory mineral analysis of all samples of clay, clay in suspension in water, and clay combined with leaf sponges, together with some comparative samples of ground soil (control soil), termite mound soil, ground water and river water controls, are provided in Table 1. Data are mg/kg for solids, ppm for water samples. Mean and range of values for each of the 7 sample groups are summarized in Table 2, together with together with 3 additional groups (RF, CP, and all other species) derived from earlier work [3] for comparative purposes.

**Table 1:** Results of laboratory analysis (mg/kg for solid samples or ppm for water samples).

No	Details of sample	Na	K	P	Ca	Fe	Mn	Al	Cu	Mg	Cl	Location
1	Clay	107.54	786.26	146.75	668.64	5639.25	54.36	13083.39	23.76	364.01	*	G6
2	Clay	113.01	819.36	177.34	797.22	6689.44	70.59	11743.08	24.7	228.05	*	G6
3	Clay	118.99	1156.25	176.28	1073.18	7483.14	77.54	17246.82	28.29	422.16	*	G6
4	Clay	106.43	806.19	135.74	760.53	5086.92	47.92	8784.39	25.49	363.65	*	G6
5	Clay	95.7	669.89	191.07	754.98	10725.39	98.22	7742.73	25.06	369.55	*	G6
6	Clay	157.12	883.07	167.11	929.83	8192.74	456.99	9259.12	25.2	470.34	*	G6
7	Clay Waibira	269	709	*	1480	8285	505	1305	*	917	*	Waibira
8	Clay-water	203.29	59.5	7.97	134.19	65.34	5.17	9.58	0.22	47.97	*	G6
9	Clay-water	204.29	67.46	10.64	141.47	132.64	5.84	20.55	0.34	54.16	*	G6
10	Clay-water	204.86	55.52	6.09	101.38	27.26	3.28	4.03	0.14	41.17	*	G6
11	Clay-water	202.36	61.46	7.94	139.29	69.68	4.84	10.34	0.23	47.62	*	G6
12	Clay-water	196.91	54.17	6.19	98.2	39.56	3.26	6.21	0.17	40.99	*	G6
13	Clay-water	202.85	64.79	8.12	117.85	65.76	4.14	9.91	0.22	47.69	*	G6
14	Clay-water KZ	300.35	75.98	2.04	125.38	1.25	0.65	0.42	0.12	92	*	Off grid W of J
15	Clay-water KZ	302.8	75.71	2.22	125.85	1.66	0.96	0.41	0.12	92.72	*	Off grid W of J
16	Clay water	8261	1884	133	3433	6463	122	18978	42	*	*	
17	Clay water	198	1318	9	401	3188	39	1633	5	*	*	
18	Clay water	253	5.84	*	17.94	*	0.13	*	*	83.65	359	G6
19	Termite soil	51.21	1520.8	902.9	12262.35	84742.34	1677	24784.75	79.19	1383.46	*	1.1
20	Termite soil	49.86	1429.53	918.07	12327.97	83722.43	1685.84	22595.76	80.26	1380.38	*	1.1
21	Termite soil	51.78	1681.67	889.11	10965.83	88977.71	1376.74	28616.03	81.01	1475.29	*	1.1
22	Termite soil	42.31	1669.58	949.26	12789.35	86189.8	1441.4	21481.27	77.12	1444.34	*	1.1
23	Termite soil	13.18	1124.95	1086.7	2976.03	32173.02	608.48	11846.89	42.86	631.85	*	
24	Clay leaf sponge Waibira	766	8923	1177	7746	13081	571	3917	*	2906	*	Waibira
25	Clay leaf sponge Waibira	512	9810	1505	8358	14401	592	4645	*	3452	*	Waibira
26	Clay leaf sponge Waibira	91	720	49	1243	7613	582	1121	*	627	*	Waibira
27	Control ground water	0.3	0.16	*	0.1	*	0.03	*	*	0.06	6.8	G6
28	Control ground water	7	0.7	*	1.7	*	0	*	*	*	16.3	G6
29	Control ground water	115	2.6	*	4.9	*	0	*	*	*	111	G6
30	Control ground water	3	5	2	10	1	0	*	*	*	*	
31	Control soil not eaten	162.57	*	*	4418.29	33420.2	1334.94	*	*	605.25	*	block 1A
32	Control soil not eaten	157.06	*	*	3831.95	80065.2	3051.22	*	*	614.01	*	block E14
33	Control soil not eaten	149.29	*	*	1790.81	50656.34	1074.45	*	*	401.36	*	block G11
34	Control soil not eaten	146.5	*	*	936.17	53744.07	1655.79	*	*	355.04	*	road
35	Control soil not eaten	133.8	*	*	1124.79	42854.59	1763.12	*	*	360.18	*	off grid E line 1
36	Control soil not eaten	163.26	*	*	3593.27	27205	1129.37	*	*	470.83	*	block 18
37	Control soil not eaten	154.67	*	*	8997.75	27890.99	1093.27	*	*	809.69	*	block AB
38	Control river water	6.41	*	*	0	0	0.13	*	*	1.72	*	River Sonso
39	Control river water	5.69	*	*	0	0	0.16	*	*	1.54	*	River Sonso
40	Control river water	6.61	*	*	0	0	0.14	*	*	1.44	*	River Sonso
41	Control river water	6.84	*	*	0	0	0.15	*	*	1.71	*	River Sonso
42	Control river water	7.36	*	*	0	0	0.16	*	*	1.22	*	River Sonso

Key: *Na* = sodium, *K* = potassium, *P* = phosphorus, *Ca* = calcium, *Fe* = iron, *Mn* = manganese, *Al* = aluminium, *Cu = copper*, *Mg* = magnesium, *Cl = chloride*

Asterisks indicate that data were not obtained.

**Table 2:** Means and (ranges) (solid samples in mg/kg, water samples in ppm) for groups of samples.

	Na	K	P	Ca	Fe	Mn	Al	Cu	Mg
Clay	116 (96–157)	853 (670–1156)	166 (136–191)	830 (669–1073)	7303 (5087–10725)	134 (48–457)	11310 (7742–17247)	25.4 (23.8–29)	370 (228–470)
Clay-water	227 (196–302)	64 (54–76)	6.4 (2.04–10.64)	123 (98–141)	50.4 (1.25–133)	3.5 (0.65–5.84)	7.7 (0.41–20.55)	0.19 (0.12–0.34)	58 (41–93)
Termite soil	42 (13–52)	1485 (1125–1782)	949 (889–1087)	10264 (2976–12789)	75161 (32173–88978)	1358 (608–1686)	21865 (11846–28616)	72 (43–81)	1263 (632–1475)
Leaf sponges	456 (91–766)	6484 (720–9810)	910 (49–1505)	5782 (1243–8358)	11698 (7613–14401)	582 (571–592)	3228 (1121–4645)	*	2328 (627–3452)
Ground water	31 (0.3–115)	2.1 (0.16–5)	*	4.17 (0.1–10)		0.0 (0.0–0.3)	*	*	0.06
Ground soil	152 (134–163)	*	*	3258 (936–8998)	45119 (27205–80065)	1586 (1074–3051)	*	*	355 (355–810)
River water	6.58 (5.69–7.36)	*	*	*	*	0.15 (0.13–0.16)	*	*	1.53 (1.22–1.72)
RF	5038 (1095–14616)	6650 (3165–12559)	367 (58–1057)	1563 (421–4568)	128 (20–515)	425 (60–1785)	*	*	2430 (293–6586)
CP	1822 (92–14330)	10462 (603–71212)	1376 (115–11597)	5474 (1005–26620)	142 (15–618)	48 (1–645)	*	*	2067 (421–7090)
Other spp	293 (0–2455)	4073 (345–24993)	851 (59–4381)	13314 (791–154913)	649 (12–5322)	66 (6–253)	*	*	1457 (143–4114)

Key: *Na* = sodium, *K* = potassium, *P* = phosphorus, *Ca* = calcium, *Fe* = iron, *Mn* = manganese, *Al* = aluminium, *Cu = copper*, *Mg* = magnesium. Asterisks indicate that data were not obtained.

### Comparison of clay, clay-water and leaf sponges

We compared mineral content in clay (n = 7), clay-water (n = 11) and leaf-sponges (n = 3), the differences between mineral content per sample type were significant for all minerals considered. Despite the small number of samples, leaf sponges showed the highest mineral concentrations in 6/8 minerals.

### Comparison of clay, clay-water and leaf sponges containing clay

We compared mineral content in clay (n = 7), clay-water (n = 11) and clay-containing leaf-sponges (n = 3), the differences between mineral content per sample type were significant for all minerals considered. Despite the small number of samples, clay with leaf sponges showed the highest mineral concentrations in 6/8 minerals (Kruskal Wallis test, df = 2 throughout: *K* (H = 7.54, *p* = 0.015), *P* (H = 12.99, p = 0.002), *Ca* (H = 11.70, p = 0.002), *Fe* (H = 14.06, p = 0.001), *Mn* (H = 13.53, p = 0.001), and *Mg* (H = 14.14, p = 0.001)). Clay had highest values for *Al* (H = 9.33, p = 0.009) and lowest values for *Na* (H = 6.44, p = 0.04).

### Comparison of clay-water, ground water and river water

Chimpanzees regularly drank water from clay-holes but rarely did so from either puddles of water lying on the soil surface or from the free-flowing water of the R. Sonso. We compared the available mineral content of clay-water in relation to groundwater. Mineral content in river water was present in insufficient quantities to be included in these analyses. Clay-water had higher mineral concentrations than ground water in the case of (Kruskal Wallis test, df = 2 throughout, *Ca* (H = 15.66, *p* = 0.001), *Mn* (H = 13.6, *p* = 0.001), *K* (H = 8.25, *p* = 0.004), and *Na* (H = 14.16, *p* = 0.001). Other minerals were not measured in all groups.

### Comparison of clay with ground soil and termite soil.

Chimpanzees do not eat ground soil in the Budongo Forest, but they do consume both clay soil and termite soil. Termite soil had the lowest amount of *Na* (Kruskal Wallis: H = 11.91, df = 2, *p* = 0.003). This was in contrast with the other minerals tested (Kruskal Wallis test, df = 2 throughout: *Ca* (H = 13.22, *p* = 0.001), *Fe* (H = 14.29, *p* = 0.001), and *Mg* (H = 9.61, *p* = 0.001) were higher in termite soil than in ground soil or in clay.

Termite soil is broken off from the tops and sides of termite mounds (*Macrotermes* and *Pseudocanthotermes*) in the forest. Clay was compared with termite soil samples from mounds where chimpanzees had been seen feeding. A comparison between clay and termite mound soil showed that termite soil differs significantly from clay in all minerals tested (Kruskal Wallis test, df = 1 throughout: *Na* (H = 8.08, *p* = 0.004), *Ca* (H = 8.08, *p* = 0.004), *Fe* (H = 8.08, *p* = 0.004), *Mn* (H = 8.08, *p* = 0.004), *Mg* (H = 7.18, *p* = 0.007), *K* (H = 7.18, *p* = 0.007), *P* (H = 7.5, *p* = 0.006), *Al* (H = 6.37, *p* = 0.012), *Cu* (H = 7.5, *p* = 0.006). In all minerals except *Na*, termite soil had higher (often much higher) mineral concentrations than clay. *Fe*, *Mn* and *Al* were especially highly concentrated in termite mound soil (see Tables 1 and 2).

### Comparison of clay with vegetative sources of minerals

Mineral acquisition from the decaying wood of two forest tree species, *Raphia farinifera* (RF) and *Cleistopholis patens* (CP) were described in earlier papers [2,5], which also described mineral content of a variety of other species eaten by chimpanzees. A comparison was made between mineral content of clay, RF, CP, and other species.

For all minerals measured, significant differences exist between these groups (df = 3 throughout). In the case of *Na* (H = 35.73, *p* = 0.001), *Ca* (H = 28.22, *p* = 0.001), *Mg* (H = 11.35, *p* = 0.010), *K* (H = 17.68, *p* = 0.001), and *P* (H = 11.49, *p* = 0.009), clay had the lowest values. However, for *Fe* (H = 18.21, *p* = 0.001), clay had the highest values and clay also had high values for *Mn* (H = 28.28, *p* = 0.001).

### Chloride

Table 1 (second-last column) shows the results of 4 measurements of chloride (*Cl*) in water samples. All levels were low. The most chloride found was in the single sample of clay-water measured (359ppm), with lower values in the 3 ground water samples (111, 16.3 and 6.8 ppm). *Cl* concentrations varied in line with *Na* concentrations, indicating the possible presence of *NaCl*.

### Change in feeding behaviour

After the decline of RF feeding owing to the loss of RF palm trees caused by the activities of local tobacco farmers [5] there was an initial increase in CP feeding [5], and this has coincided with evidence of an increase in feeding on clay and clay-sources, see Tables 3 and 4. Table 3 shows the changes of RF and CP feeding from 2008–2014, using data from the BCFS database. In this table, SO includes clay eating and termite mound soil eating. RF feeding declined after 2009, with larger groups feeding in 2012 and 2013 than in earlier years, perhaps indicating that, with fewer trees available, more individuals were present in the groups feeding at each remaining tree. While quantitative data on frequency of RF, CP and clay consumption are not available, in Table 4 we employed the terms previously established to describe frequency of cultural behaviours. Prior to 2000 RF feeding was “customary” (commonly observed) among the Sonso chimpanzees, from 2000–2005 it reduced to “habitual” (observed repeatedly in several individuals) and after 2005 RF feeding reduced further to “present” (clearly identified but neither customary nor habitual). Over the same period, *CP* feeding changed from “habitual” to “customary”, declining to “present” after 2012. Clay feeding and clay-water drinking were “present” prior to 2005, after which they became “habitual” and then “customary”.

**Table 3:** Changes in feeding on *Raphia farinifera* (RF), *Cleistopholis patens* (CP) and all soil including clay and termite soil (SO), 2008–2014.

Year	Feed	Days (n)	Blocks (n)	Mean no feeding
2008	RF	46	11	7.9
	CP	3	3	14.4
	SO	-	-	-
2009	RF	14	10	5.3
	CP	12	9	5.3
	SO	1	1	12.7
2010	RF	4	4	6
	CP	17	19	7
	SO	1	1	14
2011	RF	5	5	5.5
	CP	7	7	9.6
	SO	3	4	9.5
2012	RF	7	4	16.3
	CP	32	25	6.8
	SO	3	3	11.6
2013	RF	6	5	13.9
	CP	17	22	8.7
	SO	4	11	15.4
2014	RF	1	1	2
	CP	3	5	17.3
	SO	3	7	4.1

**Table 4:** Frequency of *Raphia farinifera* (RF), *Cleistopholis patens* (CP) and clay, 1991–2014 (Qualitative data).

Dates	RF pith being eaten	CP dead wood being eaten	Clay eaten, clay-water drinking	Tobacco growing
1991–2000	Customary	Habitual	Present	Small scale
2000–2005	Habitual	Customary	Present	Large scale
2005–2011	Present	Customary	Habitual	Reducing, ended 2011
2011–2014	Present	Present	Customary	None

## Discussion

### Comparisons between groups of samples

Clay had the lowest concentration of *Na* of all the groups compared, suggesting that chimpanzees may not have been consuming clay for *Na*. By contrast, clay had the highest concentration of *Al*, indicating that the clay may take the form of kaolinite, an ingredient of clays eaten elsewhere for their digestive properties [6]. 6 out of 8 elements, *K*, *P*, *Ca*, *Fe*, *Mn*, and *Mg*, were all present in high concentrations in the clay-containing leaf sponges, as compared to clay obtained in clay-water or via direct consumption. One possible explanation is that leaves may contribute to the amount of these mineral elements present in the clay. Similarly, clay has in the past been found to increase the bioactive properties of leaves [7]. We show here that leaf sponges containing clay contribute minerals to the chimpanzees’ diet, and the fact that the clay is probably in the form of kaolinite may help with adsorption and digestion of these minerals in the chimpanzee gut. We suggest that the consumption of clay, in particular by the associated method of leaf sponging, is a means of supplementing minerals that are scarce in the fruit and leaves that make up the largest proportion of their diet.

Clay-water was compared with ground water and river water. The ground water took the form of puddles of accumulated water lying on the ground where no clay was evident but may have been underlying the water. Such puddles might be the result of rainfall or of river water, and were sometimes sponged by chimpanzees. In all cases clay-water had higher mineral concentrations than ground or river water. This may help to explain the focus of the chimpanzees on certain waterholes beneath trees where the soil consisted of clay. In such places there are high concentrations of minerals not found in other sources of water in the forest.

Clay was compared with ground soil, which, at Budongo, chimpanzees do not eat. This is in contrast with the situation at Kibale Forest, where ground soil is regularly eaten [8]. Samples of ground soil were taken at places chosen at random in the forest, and to ensure that soil rather than decomposing leaf litter was collected, they were taken from 10cm below the forest floor. Ground soil was found to have high concentrations of *Fe*, *Mn* and *Ca*. The high concentration of *Ca* may be the result of organic matter permeating the soil [8]. Although the difference was not significant in the case of *Na*, it was in the same direction, i.e. higher in ground soil than in clay. In the case of *Mg* there was no difference. One reason for this could be that, rather than the particularly high concentration of minerals, it is the specific, fine-grained consistency of clay that attracts chimpanzees to eat it, thus obtaining a spectrum of minerals in digestible form.

Chimpanzees in Budongo Forest eat the soil of termite mounds, of at least two species. When compared with clay, termite mound soil contained surprisingly and significantly higher concentrations of all minerals except *Na* in termite mound soil compared to clay. For *Fe* and *Al*, there was as much as a tenfold difference in concentration. However, *Na* marked a complete departure from this pattern, with a very low concentration in termite soil, similar to that of ground water (see Table 2). Low levels of *Na* and of *Cl* have also been reported from termite soil at Mahale [9]. However, in contrast with the findings of Mahaney et al [9], termite soil eating does not appear to be self-medication for diarrhoea as it is not associated with signs of diarrhoea in Budongo Forest.

### Increase in clay eating over time

Formerly the Budongo chimpanzees had regular access to a high quality source of *Na*, the decaying pith of *Raphia farinifera* (RF), a palm tree growing in Swamp Forest. This species was all but eliminated by the activities of tobacco farmers who used its leaf stems for tying and curing tobacco leaves [2]. A secondary source of *Na* then became prominent in the diet of the chimpanzees, the dead wood of *Cleistopholis patens* (CP) [5]. Clay had less *Na* than RF or CP and contained approximately the same (small) amount as was present in the other species tested. These low levels suggest that clay has probably not taken the place of RF or CP for obtaining *Na*. It may however be a source of other minerals that are now being used more extensively than in the past.

### Mineral requirements of primates

Clay is eaten by black and white colobus monkeys (*Colobus guereza*) in Budongo Forest and also at Kanyawara, Kibale Forest, where it provides a range of chemical elements albeit mostly in low quantities [10, 11]. There is a potential sodium deficiency in Kibale colobus monkeys, indicated by urine drinking and consumption of swamp plants [10, 11]. The sodium and iron content of the majority of foods eaten by these (mainly leaf-eating) monkeys is very low [11]. Average mineral intake by red colobus was 193 mg/kg and for black and white colobus was 189 mg/kg [12]. However, only one of 8 colobus groups studied selected plants for their sodium content, indicating that levels of *Na* present in plants may be below the animals’ taste threshold. Levels of sodium found were < 300 mg/kg [12]. National Research Council requirements for this species in captivity are 2000 mg/kg, although requirements for wild colobus are not known [13]. Figs showing low levels of *Na* and *Fe* intake from the normal diet of cercopithecine monkeys (*Na* 168–170 mg/kg, *Fe* 142–170 mg/kg) and chimpanzees (*Na*162 mg/kg, *Fe* 154 mg/kg), based on dietary data from Wrangham et al [12], indicate that dietary supplementation of these minerals can be expected. The taste threshold of wild chimpanzees for *Na* (in the form of *NaCl*) and other minerals is not known. Studies of taste perception in captive chimpanzees indicate that the levels of *Na* found in clay in the present study probably fall below the level of taste perception [14,15,16]. However, *Fe*, which as we have shown reaches high levels in clay and especially in termite mound soil, may be a discernible taste as termite soil eating has been described as “rolling pieces of it in their [chimpanzees’] mouth for long periods of time” (Lamon, pers. comm.).

The Sonso chimpanzees, regularly monitored by the BCFS veterinarian team, show no overt, behavioural or physical signs of hyponatremia or other signs of mineral deficiency (Asiimwe, C., pers. comm.). Hunting by chimpanzees of *Colobus guereza* occurs regularly, and, besides a valuable source of proteins, the meat obtained from such hunts is no doubt a valuable source of *Na* and other minerals for those chimpanzees who manage to access a piece of the meat. Clay, though not containing much *Na*, is rich in other minerals and it may be important in contributing other minerals, such as *Fe*, to chimpanzee health. Rode et al [11] report that the vegetative diet of primates in Kibale Forest is deficient in *Fe*, and Mahaney et al [8,9] consider that the high levels of *Fe* they found in soils eaten by chimpanzees at Kibale offered the most likely stimulus for geophagy. As shown in this paper, *Fe* is a prominent constituent of clay and ground soil. Energy expended in obtaining clay is likely substantially less than energy expended in obtaining meat, perhaps making it a more attractive dietary option than hunting for some chimpanzees.

### Digestive benefits and detoxification

A commonly known explanation for eating clay is that the mineral-binding properties of the fine particulate structure of clays (kaolinite in particular) aid in digestion, counteract over-acidity and toxins, and aid absorption of nutrients in the gut [9, 17, 18]. The presence of a high level of aluminium in the clay consumed by Budongo chimpanzees (but not in clay-water, Table 2) indicates that the clay was in large part kaolinite. It appears that clay, besides mineral supplementation, is important in detoxification, especially of tannins and other antifeedants [17, 18]. Condensed tannins are present in many chimpanzee food items, both fruits and leaves, especially mature leaves [19]. Chimpanzees are able to tolerate a high tannin:fructose ratio (66%) [20], but may nonetheless need to neutralise the antifeedant effects of tannins. For black and white colobus monkeys, which frequently eat clay in Budongo Forest, clay may also be an important source of detoxification.

The adsorption of minerals by clay has led to its consumption by humans for mineral supplementation, in particular for *Na* and *Fe* [21]. Release of a number of mineral elements, especially iron, from clay (kaolinite) in the presence of tannic acid has been demonstrated [21], with mineral release increasing with increased acidity from pH 4 to pH 1. Local women in the communities around Budongo consume forest clay mixed with water for stomach problems and during pregnancy (Gideon Monday pers. comm.). Wild orangutans and gorillas add mineral supplements to their diet. A study of wild orang-utans in Borneo found them visiting natural licks, where they obtained seepage soil water in preference to actual soil. Mineral analysis showed the presence in the water of calcium, magnesium, potassium and sodium, the latter being especially prevalent [22]. Mountain gorillas consume dead wood, which has been found to be rich in sodium [23].

### Clay, clay-water, and leaves

Sodium and other positively charged mineral ions (cations) such as aluminium, calcium, iron, magnesium, and potassium become attached to clays such as kaolinite in the presence of water. This pore-fluid can then leach out into surrounding water that, as a result, becomes increasingly mineral-rich. The concentrations of these minerals in the clay and clay-water is determined by the processes of flocculation and dispersion of the mineral atoms present [24]. Chimpanzees at Budongo have discovered this source of minerals, which they are able to access with the use of leaf sponges. Leaves also contain the above mentioned cation elements. However, chimpanzees do not always use leaves to access minerals. For example, the Kibale chimpanzees are not described as using leaf sponges to access clay-water [8]. We have shown that clay consumed with leaf sponges provides higher mineral concentrations than clay-water or clay.

The use of leaves by wild chimpanzees in Kibale Forest has given rise to the hypothesis that certain leaf species, e.g. *Trichilia sp*, have bioactive constituents that are more readily released in the presence of clay than when clay is not present [7,25]. This would provide an additional function of leaves from what we describe in the present study; here it seems that the chewing and sucking of leaves increases the amount of minerals obtained from clay and clay water. Leaf sponges are also made by chimpanzees for drinking river water and water from tree-boles, and in these cases there is a preference in Budongo Forest for leaves of the genus *Acalypha* spp., not part of their normal leaf diet [26]. A study of leaf-assisted drinking at Budongo recorded that in 78/111 recorded cases the leaves used were of the genus *Acalypha*, although 8 other species of leaves were also used [26]. In Kibale Forest chimpanzees show a preference for making leaf sponges for drinking water out of *Acalypha ornata*, *Aframomum* sp., and *Bosqueia phoberos* [7]. Data from both forests indicate selection for particular leaf species and possibly thus possibly selection for particular biochemical properties of those leaves [7]. The use of particular leaf species by chimpanzees, together with clay to neutralise gut toxins, release bioactive nutrients, and possibly enhance flavour, has recently been suggested [27].

Thus, in addition to the use of leaves of certain species for detoxification purposes, and the use of clay for the purpose of mineral supplementation, there may also be an interaction of clay and leaves that increases the amount of certain mineral elements obtained from the clay. Use of leaf sponges may thus increase the amount of *Na* in clay-water, and this could also explain why the relatively low levels of *Na* in clay are higher in surrounding clay-water. However, we should note the small sample size, so these results must be considered as tentative.

## Conclusions

We conclude as follows:

1. Clay is potentially eaten for its mineral content as well as having detoxification properties.
2. The high values of Al in clay indicate that it probably takes the form of kaolinite.
3. The use of leaves in the form of leaf-sponges to obtain clay not only increases mineral acquisition but may also bring about an interactive effect of clay and leaves to further increase the quantity of minerals obtained.
4. Termite mound soil contains particularly high concentration of soil minerals, which may make it attractive to chimpanzees. However, sodium is an exception and is not found in concentrated form in termite mound soil.
5. There is suggestive evidence that following the decline of feeding on decaying *Raphia farinifera* pith, there has been a rise in feeding on clay.

## Supporting Information

S1 Video Feeding on clay, juvenile male, (video Anne Schel, #14382, 30 November 2011). (MP4)

S2 Video Drinking at waterhole, 3 adult females, 1 adult male, 2 subadults, 3 juveniles/infants (video Cat Hobaiter, #14382, 30 November 2011). (MP4)
